# The origins of malaria artemisinin resistance defined by a genetic and transcriptomic background

**DOI:** 10.1038/s41467-018-07588-x

**Published:** 2018-12-04

**Authors:** Lei Zhu, Jaishree Tripathi, Frances Maureen Rocamora, Olivo Miotto, Rob van der Pluijm, Till S. Voss, Sachel Mok, Dominic P. Kwiatkowski, François Nosten, Nicholas P. J. Day, Nicholas J. White, Arjen M. Dondorp, Zbynek Bozdech, Tracking Resistance to Artemisinin Collaboration I, Aung Pyae Phyo, Elizabeth A. Ashley, Frank Smithuis, Khin Lin, Kyaw Myo Tun, M Abul Faiz, Mayfong Mayxay, Mehul Dhorda, Nguyen Thanh Thuy-Nhien, Paul N. Newton, Sasithon Pukrittayakamee, Tin M. Hlaing, Tran Tinh Hien, Ye Htut

**Affiliations:** 1 School of Biological Sciences, Nanyang Technological University, Singapore, 637551 Singapore; 2 Mahidol-Oxford Tropical Medicine Research Unit, Faculty of Tropical Medicine, Mahidol University, Bangkok, 10400 Thailand; 3 Centre for Tropical Medicine and Global Health, Nuffield Department of Medicine Research, University of Oxford, Oxford, OX3 7LF UK; 4 Medical Research Council (MRC) Centre for Genomics and Global Health, University of Oxford, Oxford, OX3 7BN UK; 5 Wellcome Trust Sanger Institute, Hinxton, CB10 1SA UK; 6 Swiss Tropical and Public Health Institute, Basel, 4051 Switzerland; 7 University of Basel, Basel, 4001 Switzerland; 8 Columbia University Medical Center, Columbia University, New York, 10027 USA; 9 Shoklo Malaria Research Unit, Mahidol-Oxford Tropical Medicine Research Unit, Faculty of Tropical Medicine, Mahidol University, Mae Sot, 63110 Thailand; 10 Department of Medical Research, Naypyitaw, 15011 Myanmar; 11 Defence Services Medical Research Centre, Naypyitaw, 15011 Myanmar; 12 The Malaria Research Group and Dev Care Foundation, Dhaka, 1209 Bangladesh; 13 Laos–Oxford–Mahosot Hospital–Wellcome Trust Research Unit, Mahosot Hospital, Vientiane, 989 Laos; 14 Oxford University Clinical Research Unit, Hospital for Tropical Diseases, Ho Chi Minh City, 710400 Vietnam

## Abstract

The predisposition of parasites acquiring artemisinin resistance still remains unclear beyond the mutations in *Pfk13* gene and modulation of the unfolded protein response pathway. To explore the chain of casualty underlying artemisinin resistance, we reanalyze 773 *P. falciparum* isolates from TRACI-study integrating TWAS, GWAS, and eQTL analyses. We find the majority of *P. falciparum* parasites are transcriptomically converged within each geographic site with two broader physiological profiles across the Greater Mekong Subregion (GMS). We report 8720 SNP-expression linkages in the eastern GMS parasites and 4537 in the western. The minimal overlap between them suggests differential gene regulatory networks facilitating parasite adaptations to their unique host environments. Finally, we identify two genetic and physiological backgrounds associating with artemisinin resistance in the GMS, together with a farnesyltransferase protein and a thioredoxin-like protein which may act as vital intermediators linking the *Pfk13* C580Y mutation to the prolonged parasite clearance time.

## Abstract (web summary)

Mechanisms underlying increasing artemisinin resistance of *Plasmodium* in Southeast Asia remain unclear. Here, Zhu et al. integrate TWAS, GWAS and eQTL analyses for 773 *P. falciparum* isolates and identify genetic and transcriptomic backgrounds to artemisinin resistance.

## Introduction

P*lasmodium falciparum*, the causative agent of the most severe form of human malaria is once again threatening human health around the world^1^. After the largely successful world-wide elimination efforts that reduced malaria-related mortality and morbidity over the last decade, resistance of *P. falciparum* to the currently used artemisinin-based chemotherapeutics presents a new challenge for the future^2,3^. *P. falciparum* strains resistant to artemisinin were first reported in western Cambodia in 2009^4^, and was subsequently detected in other parts of Southeast Asia^5^ including regions near India^6^. It is generally understood that an import of artemisinin resistance to Africa (presumably via India) will have a devastating effect, causing millions of deaths and other severe social and economic setbacks^7,8^. Hence, it will be crucial to understand all biological aspects of artemisinin resistance, particularly its emergence and spread^9,10^. This knowledge will not only help to manage the current situation but possibly also to guide future deployments of new malaria chemotherapies.

The current phenotype of artemisinin resistance is associated with nonsynonymous single nucleotide polymorphisms (SNPs) in the sequence encoding the Kelch13 (PfK13) propeller domain of *P. falciparum*^11^. The causative *pfk13* SNPs were detected in *P. falciparum* isolates in western Cambodia as early as 2009 and in other regions of the Greater Mekong Subregion (GMS) in the following years^12,13^. Up to 26 nonsynonymous SNPs of the *pfk13* gene, linked to artemisinin resistance, were identified in *P. falciparum* isolates from Southeast Asia between 2009 and 2014^5,9,14^. Using haplotype analyses, Takala-Harison et al.^15^ demonstrated that at least 12 of the *pfk13* SNPs emerged independently in several regions of Laos, Myanmar and Cambodia and spread locally. Only two of the identified mutations, C580Y and Y493H, exhibited a long distance transmission pattern from Cambodia to Vietnam. C580Y has only recently reached a state of fixation in many parts of the GMS, being transmitted in the context of two distinct long-range haplotypes, one of which originates in Cambodia and the other in Myanmar^16^. Hence, the currently spreading phenotype of artemisinin resistance appears to be a consequence of a selection process that maximizes survival and transmission of the most efficient genotype/genetic background of *P. falciparum* parasites.

Only limited information exists about the putative genetic and transcriptomic backgrounds supporting the *pfk13* mutations in conferring artemisinin resistance. First, the malaria parasite cohort in the western Cambodian province, Pailin, the epicenter of artemisinin resistance, exhibits a highly unusual structure characterized by skewed allele frequency spectra and high haplotype homozygosity; indicating a strong founder effect^17^. Second, GWAS studies identified at least five SNPs that are strongly associated with artemisinin resistance and presumably contribute to the resistance-driven phenotype^18^. Third, an additional set of SNPs co-segregated with the *pfk13* SNPs (particularly with C580Y) across the GMS between 2001 and 2014^19^. Finally, artemisinin resistance is associated with a specific transcriptional profile that is characterized by induction of the unfolding protein response (UPR) and deceleration of the intraerythrocytic developmental cycle (IDC)^20,21^.

To explore this further, we carried out a large-scale bioinformatics analysis combining whole genome sequences and transcriptomes from 773 *P. falciparum* field isolates collected across seven countries in Southeast Asia between 2011 and 2013^5,18,20^ during the Tracking Resistance to Artemisinin Collaboration (TRACI). Utilizing eQTL analysis, we captured two distinct physiological and genetic makeups that are different between the *e*- and *w*-GMS. Furthermore, using an eQTL analysis, we identified distinct set of genetic/physiological background for artemisinin-resistant parasites in *e*-GMS and *w*-GMS with key proteins, the farnesyltransferase protein of PF3D7_1242600 and the thioredoxin-like protein of PF3D7_0717900, which may mediate the *Pfk13* C580Y effect on artemisinin-resistant parasites. Overall, we outline new genetic and transcriptional factors that contribute to the emergence and spread of artemisinin resistance in the GMS and beyond.

## Results

### Population transcriptomics of the GMS *P. falciparum*

**Fig. 1:**
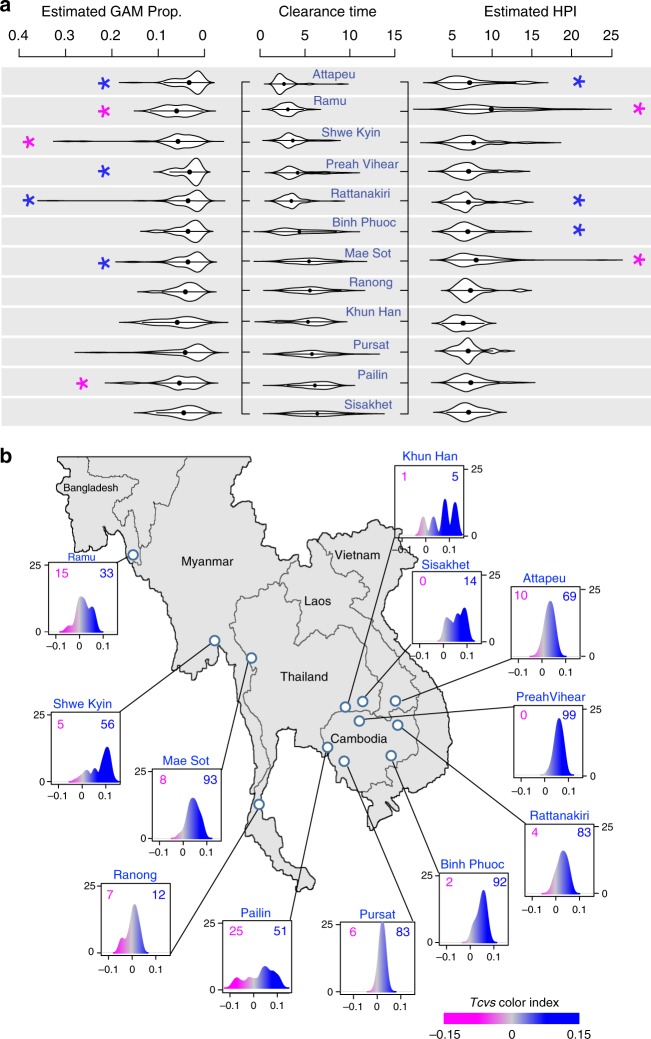
*P. falciparum* transcriptome in the GMS. **a** Violin plot representing the distribution of estimated gametocyte proportion (GAM Prop.; left) and asexual parasite age (HPI; right) for all the GMS sites arranged in order of median parasites clearance time (in response to artemisinin treatment) (middle) from high to low. Black dot within violin indicates the statistical mean of each category. Magenta (blue) asterisks denote significantly high (low) values at *p*-value < 0.05 (Wilcoxon rank-sum test). **b** Distribution of *Tcvs* is displayed in density plot to show the local convergence of parasite transcriptome at each TRACI-study sites of the 773 isolates. The color from magenta to gray to blue represents the *Tcvs* scores from negative to zero to positive. The isolates number with *Tcvs* > 0 (blue number) and *Tcvs* ≤ 0 (magenta number) are shown together with the density plot at each site. The geographic map of Southeast Asia is originally downloaded from [https://freevectormaps.com/world-maps/southeast-asia] and modified using Photoshop

Initially, we re-analyzed the TRACI *P. falciparum* transcriptome dataset of 773 isolates^20^ in order to dissect transcriptional changes that reflect physiological states of individual parasites within the patients. Principal component analysis (PCA) revealed that the major transcriptional differences among the field isolates are reflections of the parasite IDC progression (the 1st and 2nd PCs) and gametocytogenesis (the 1st and 3rd PCs, Supplementary Fig. 1a, b). Hence, we estimated the dominating IDC stage (hour post invasion, HPI) and the proportion of the sexual forms (gametocytes, GAM) in each parasite sample using a mathematical model based on maximum likelihood with the reference in vitro transcriptional profiles of the IDC^22^ and stage IV/V gametocytes (Methods). This allowed us to estimate the life cycle representation (HPI and GAM) of *P. falciparum* parasites in the peripheral blood samples at the individual TRACI field sites. We found that the isolates collected in Pailin, Shwe Kyin, and Ramu presented increased GAM proportions (median > 5%). Conversely, parasites from Attapeu, Preah Vihear, Rattanakiri, and Mae Sot exhibited decreased GAM proportions (median < 2%) (Fig. 1a). Also, the parasite cohorts from Attapeu, Ratanakiri and Binh Phuoc were skewed towards younger ring stages (median HPI ~6.6–7 h) while the parasites from Mae Sot and Ramu seemed older (median HPI ~7.8–8.5 h). Interestingly, the increased GAM proportion coincided with a high occurrence of artemisinin resistance in Pailin, while a decreased GAM proportion and advanced HPI coincided with the high level of artemisinin resistance in Mae Sot.

In the next step, we adjusted the transcriptome dataset for the HPI and GAM biases using a linear regression model by extracting residual expression values (Methods). This allowed us to construct a transcriptional dataset that reflects the actual adaptation of the individual parasites to its host environment. As expected the PCA of these adjusted transcriptomes showed much more dispersed GAM and HPI distribution (Supplementary Fig. 1c, d) and revealed additional components that are associated with the individual site cohorts (Supplementary Data 1). Inspecting the standard deviation of the corrected expression we observed that greatest transcriptional variability is generally exhibited by genes related to host-parasite interactions, such as host cell remodeling, cytoadhesion, antigenic variation, and host cell invasion; and also to metabolic and cellular processes, such as lipid metabolism, glycolysis, protein trafficking, and endoplasmic reticulum (ER) stress (Supplementary Fig. 2). Regarding the genes encoding surface antigens, we particularly detect most variable expression for the *phist* gene family, *rifins*, *stevors*, knob-associated histidine-rich protein (*kahrp*), mature-parasite-infected erythrocyte surface antigen (*mesa*), ring-infected erythrocyte surface antigen (*resa*), *surfins,* and genes encoding Maurer’s cleft components. This indicates the pivotal role of these genes in general adaptation of *P. falciparum* parasites to their host environment at the regional level.

To investigate transcriptome regionalization, we measured transcriptome differentiation within each geographic location/site for each individual parasite cohort. For this, we developed the Transcriptome Local Convergence index (*Tcvs*), which is derived as a normalized ratio of the transcriptome distance between local and global/distal sample sets using the transcriptome dataset adjusted for HPI and GAM (Methods). The *Tcvs* showed a marked regional conservation of transcriptomes with the majority (89%) of GMS *P. falciparum* isolates forming positive *Tcvs* peaks at individual sites (Fig. 1b, blue and Supplementary Data 2). The highest local convergence was observed in Preah Vihear (*p*-value = 1e–16, Mann–Whitney *U* test of *Tcvs* against the entire GMS), Shwe kyin (*p*-value = 5e–13, Mann–Whitney *U* test) and Binh Phuoc (*p*-value = 2e–4, Mann–Whitney *U* test). On the contrary, a smaller proportion of the GMS *P. falciparum* isolates (11%) exhibited local divergence from their counterparts (Fig. 1b, magenta). The most significant divergence was observed in Pailin where 32% of the isolates presented zero or negative *Tcvs* (*p*-value = 2.8e–10, χ^2^ test against the expected frequency of 11%); and Pursat where *Tcvs* scores are the most close to zero. A Gene Set Enrichment Analysis (GSEA) revealed that the geographical transcriptome convergence is driven predominantly by genes involved in ribosome biogenesis and structure, ubiquitin-dependent protein catabolic process, translation, RNA processing, DNA damage repair, glycolysis, redox homeostasis, and phospholipid metabolism. Conversely, transcriptome divergence seems to be determined by genes involved in cytoadhesion, ER-associated protein catabolic process, GPI anchor biosynthesis, DNA replication, and exported proteins such as the PHIST gene family. Significant transcriptome diversion in Pailin and Pursat was also found to be associated with genes in involved in rosette formation, cellular redox homeostasis, ER stress, fatty acid biosynthesis, mitosis, and merozoite invasion (Supplementary Data 3).

There was a high level of genetic relatedness of the individual cohorts in all sites including those with the transcriptionally diverse parasites such as Pailin and Pursat and to some degree Ranong and Ramu (Supplementary Fig. 3a). This suggests that while some of the observed transcriptional differences are linked to the local genetically driven adaptations, others (especially those with the greatest variations) are related to other processes, such as stress response which is discussed in the section of eQTL mapping and association studies of artemisinin resistance. However, the founder-like (sub)populations defined genetically in some of the study sites^17^ do not correlate directly with the transcriptional differences (Supplementary Fig. 3b-f). This is consistent with our initial hypothesis suggesting that (some) transcriptional variations might have preceded the genetic diversion within the individual sites. Some epidemiological aspects such as drug resistance could drive the local transcriptional diversion. This is demonstrated by the increased diversion (*Tcvs* < 0) of Pailin parasites that carry the piperaqine resistance marker, amplification of plasmepsin II–III^23^, that reached 50% prevalence in this (sub)regions in 2011–2013 (Supplementary Fig. 3g)^3^.

### Diverged physiological backgrounds in the GMS

**Fig. 2:**
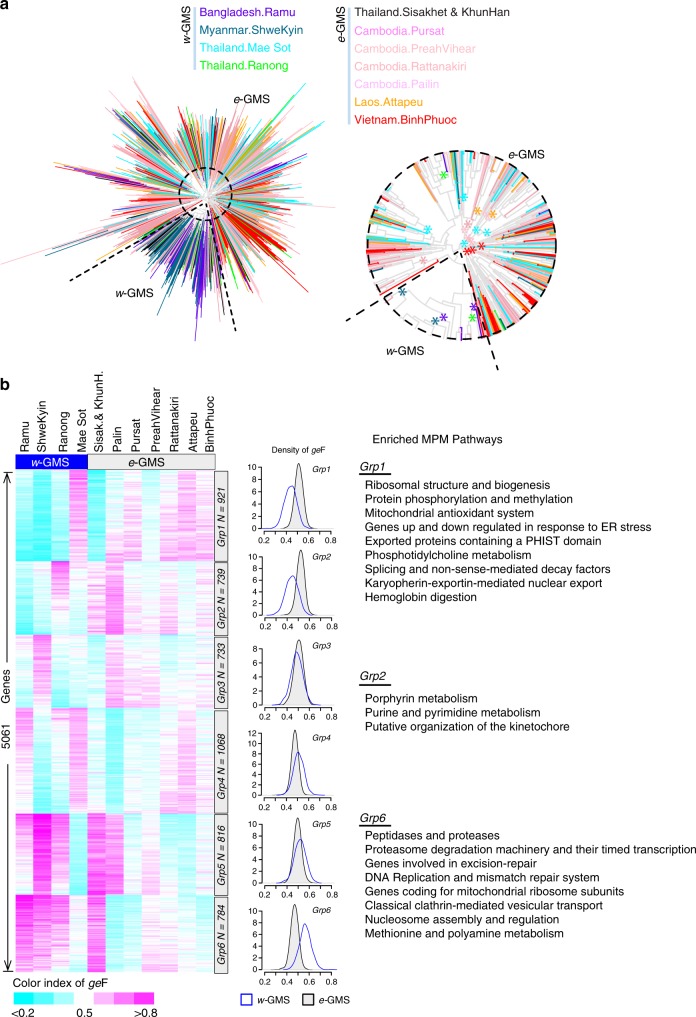
Genetic and transcriptomic population structure of *P. falciparum* isolates by geography. **a** Neighbor-joining tree showing parasite transcriptome population structure across GMS using HPI and GAM adjusted expression data. A magnified view of the branch structure is shown in the circular subpanel below the tree. Colored asterisks on branching point indicate an over-representation of cohort isolates from the respective sites (*n* > 10 and *p*-value < 0.05, hypergeometric test). The black dotted line is drawn at the main branch separating the majority of the *w*- and *e*-GMS samples. **b** Heat map of *ge*F for 5061 genes across 11 field sites of the GMS. The length of scale bar is 0.1 as shown in bottom. Six groups (*Grp1* to *Grp6*) were used to characterize the expression differences between *w-* and *e-*GMS which was obtained by K-mean clustering based on Euclidean distance. Density plot of *ge*F was generated to compare expression prevalence of each group in *w*-GMS (blue curve) and *e-*GMS (black curve shaded in gray) isolates. Enriched MPM pathways for *Grp1*, *Grp2*, and *Grp6* shown on the right were determined by hypergeometric test at *p*-value < 0.05. Full list of the enriched pathways are shown in Supplementary Data 4

Previous genetic studies have suggested a strong separation of *P. falciparum* populations between the western and eastern parts of Southeast Asia (*w*-GMS and *e*-GMS)^18^. The *w*-GMS includes Bangladesh, Myanmar, western and southern Thailand, while the *e*-GMS includes the core GMS including Cambodia, Laos and their border regions with Thailand and Southern Vietnam. Multidimensional scaling analysis of the genotypes of 773 *P. falciparum* isolates confirmed this geographical separation (Supplementary Fig. 4 and Methods). Moreover, phylogenetic tree construction based on pairwise transcriptome distances also revealed a general separation between the *w*-GMS and *e*-GMS *P. falciparum* parasites, albeit to a lesser degree (Fig. 2a). The *w*-GMS sub-branch included the majority of *P. falciparum* isolates from Bangladesh and Myanmar (72%) but also a small proportion (12%) of *e*-GMS isolates. The *e*-GMS branch contained 85% of the parasites from Cambodia, 84% from Thailand, 99% from Vietnam, and 100% from Laos. Peculiar population stratification was formed by the parasites from the western border regions of Thailand (Mae Sot), which were genetically close to *w*-GMS populations but transcriptionally intermixed with *e*-GMS populations almost equally.

To parse the physiological differentiation, we designed an index of gene expression Frequency (*ge*F) denoting the proportion of isolates with increased mRNA levels compared to the average level across the GMS based on the HPI and GAM adjusted data. K-means clustering (*k* = 6) of *ge*F on 5061 genes mainly presented two patterns (Fig. 2b). First, isolates from the *e*-GMS showed increased *ge*F for genes involved in early ribosome biogenesis, hemoglobin digestion, nuclear import/export, and mitochondrial antioxidant system (*Grp1*), as well as purine/pyrimidine metabolism and organization of the kinetochore (*Grp2*). On the other hand, the *w*-GMS isolates showed significantly higher *ge*F values for genes involved in DNA mismatch repair and DNA replication complex formation, nucleosome assembly, and proteasomal degradation (*Grp6*). Similar *ge*F values were observed in *e*-GMS and *w*-GMS isolates for genes in *Grp3, Grp4, and Grp5* (Supplementary Data 4). These results suggest that the genetic differences in geographically delineated *P. falciparum* populations may be converted into transcription-driven physiological states and are characterized by the probability of transcriptional upregulation/downregulation of individual genes potentially reflecting the adaptation of malaria parasites to varying epidemiological aspects. These processes may reflect such global regional groupings (e.g., *w*- and *e*-GMS) but also potentially more localized adaptation processes within each site that could be masked in this analysis by the global sample grouping. Data provided in this manuscript could facilitate future studies within the individual site-specific cohorts to uncover the transcriptomic-driven adaptation in a greater detail.

### eQTL mapping

**Fig. 3:**
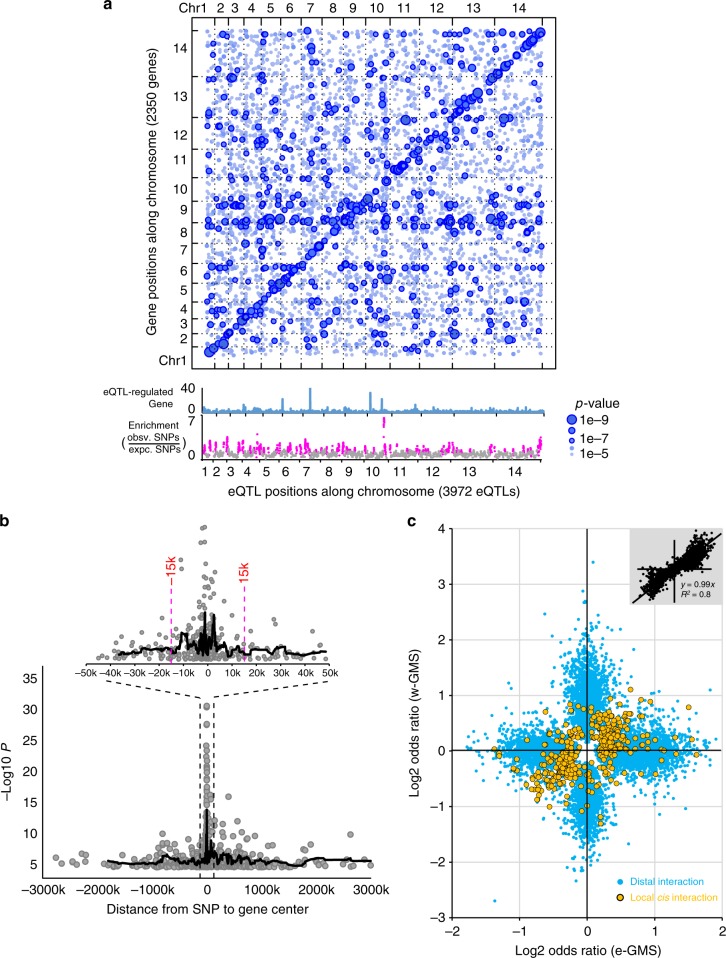
eQTL analysis for *P. falciparum* isolates in the GMS. **a** Scatter plot of 5575 high-confidence SNP-expression linkages with 2350 gene expression (*y axis*) against 3972 eQTLs (*x axis*). The statistical significance of each linkage is represented by the size of dot and the intensity of blue. The bar graph represents target genes for each eQTL. The scatter plot below represents eQTL enrichment along the chromosomes. The enrichment score was calculated as a ratio of observed and expected eQTL counts within a 50 kb window. The magenta dots denote eQTL hotspots. **b** Intra-chromosomal linkages are plotted as the significance (log transformed *p*-values) against the distance (between eQTL and target gene) for each linkage. The black lines represent average curves for a sliding window of 20 eQTLs. **c** Differential eQTL effects in isolates of (sub)regions of the GMS. LOD values are plotted for the *w*-GMS eQTLs against the *e*-GMS eQTLs where yellow dots stand for the local *cis* linkages and blue for the distal. The top right inset represents LOD values resulted from sub-sampling analysis of the 5575 high-confidence linkages in *e-*GMS (Methods)

Next, performed eQTL analyses to understand the genetic determinants of physiological variations in the two *P. falciparum* GMS populations. Essentially, we mapped the expression of 5061 genes onto 28,594 high-confidence SNPs with minor allele frequency (MAF) > 0.01. The SNP dataset included 17,589 (62%) SNPs from protein-coding regions and 11,005 (38%) SNPs from introns or intergenic regions. The expression data was normalized to minimize non-genetic component effects through the removal of top-selected PCs using linear regression analogously to other reported eQTL analyses in other eukaryotic systems^24–28^. Finally, the eQTL mapping was carried out in two independent panels for *w*-GMS and *e*-GMS populations with 229 and 544 genotyped parasites represented in the each group (Methods). Overall, we uncovered 8,720 SNP-expression linkages in *e*-GMS (Supplementary Data 5) and 4537 linkages in *w*-GMS (Supplementary Data 6) at *p*-value < 1e–05 (FDR < 0.25 by expression permutation). For subsequent functional analysis, we selected the highest confidence 5575 linkages (Supplementary Data 7) that showed no contradictory associations between *e*-GMS and *w*-GMS (Fig. 3a and Methods). These 5575 linkages represented associations between 3972 polymorphisms (eQTLs) and 2350 transcripts. We found 47% of the eQTLs appear to be physically linked, being enriched in up to 58 chromosomal clusters (Fig. 3a and Supplementary Data 8). The strongest clustering was observed on chromosome 10 (134,0673 bp to 1,511,622 bp) with 171 eQTL occurrences and an over-representation of intergenic eQTLs (*p*-value = 3e−07, binomial test against overall SNPs). However, only 3% of eQTLs associated with more than three transcripts. The top eQTL had 41 putative transcript targets and it was located at 730, 268 on chromosome 7 within an eQTL cluster (Pf3D7_07_v3: 706,086–772,669 bp) representing a nonsynonymous SNP producing a L721F substitution for gene PF3D7_0716700. In the future, it will be interesting to study these eQTLs hotspots for their potential functions as key transcriptional regulatory mechanisms.

Next, we categorized the 5575 linkages into local and distal linkages (Supplementary Table 1) at the cutoff of 15 kb between an eQTL and its regulated target(s) based on the stronger linkages (Fig. 3b) and a higher gene density (Supplementary Fig. 5) observed within this range. The interval of 15 kb within the *P. falciparum* genome contains on average three or less genes and thus linkages within this window may reflect direct physical contacts of DNA regulatory elements. This assumption is based on the recently revealed 3-D architecture of the *P. falciparum* genome, which appear to reflect a folded chromosomal structure that strongly promotes local linkages^29,30^. This criterion yielded 360 local linkages (7%), which linked 344 SNPs (local eQTLs) to transcriptional variations of 171 genes. Among the local eQTLs, 134 fell into the adjacent intergenic regions, from which 77 were located directly within the potential promoters upstream to their target genes. The intergenic eQTLs were more frequently linked to genes encoding PHIST proteins (PHISTa, PHISTb, PHISTc, RESA, LSAP2), protein kinases and proteins related to protein phosphorylation or trafficking (*p*-value < 0.05, hypergeometric test). Nonetheless, the identified eQTL set was dominated by distal linkages. To better capture the potential effects of distant regulatory elements like long-range enhancers, we further separated the distal linkages into 507 distal *cis* (intra-chromosomal) and 4708 distal *trans* (inter-chromosomal) linkages. The 507 distal cis linkages linked 483 SNPs (distal *cis* eQTLs) to 337 genes that were enriched for genes coding chaperones and their regulators as well as ER stress and genes related to peptidases/proteases. Similar functional biases were y observed in the 4708 distal *trans* linkages which linked 3381 SNPs (*trans*-eQTLs) to 2147 genes. Moreover, the rotein-coding distal *trans*-eQTLs preferentially regulated genes of basic metabolic and growth-related cellular functions, such as DNA repair, protein synthesis and folding, pyruvate metabolism, and transcription regulation and RNA processing. On the other hand, genes involved in autophagy, transcriptional regulation, mRNA processing, and redox processes were mainly associated with intergenic distal *trans*-eQTLs (for details see Supplementary Data 9).

Crucially, there appears to be major differences in the distal eQTLs obtained from *e*-GMS as compared to *w*-GMS. This is demonstrated by a mutually exclusive pattern of the distal eQTL regulations represented by the logarithm odds ratios (LOD) differentiation between *e*-GMS and *w*-GMS (Fig. 3c, blue). Essentially, no distal linkages manifesting on one side of the GMS (west or east) exhibited a similar association on the other. This is in contrast to the local linkages for which concordant associations can be observed on both sides of the GMS (Fig. 3c, yellow). Note, the LOD scores differentiation of the distal linkages is not a result of sample size or PCs normalization as randomized sub-samplings resulted in significantly high correlations (Fig. 3c, inset and Methods). Importantly, the mutually exclusive pattern of the *e*- and *w*-GMS segregation was observed for both *cis*- and *trans*-acting distal eQTLs, separately (supplementary Fig. 6). This pattern is also preserved across multiple MAF thresholds and eQTL significance cutoffs. Overall, this dramatic difference in the distal eQTL profiles further strengthens the above-mentioned concept of the division of *P. falciparum* populations between *w*-GMS and *e*-GMS showing even a stronger separation than those demarcated by the genomic and transcriptomic profiles alone.

### Association studies of artemisinin resistance

**Fig. 4:**
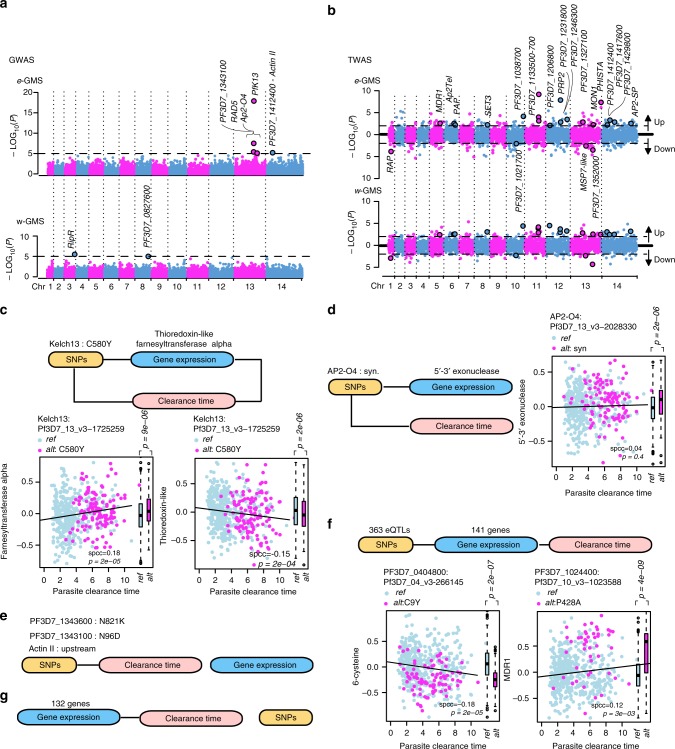
Integrative analysis of artemisinin-resistant parasites. **a** Manhattan plot of GWAS for artemisinin resistance in *e*-GMS (upper) and *w*-GMS (lower). SNPs passing *p*-value < 1e−5 are displayed in black circles above dot lines. **b** Manhattan plot of TWAS for artemisinin resistance. Upregulated and downregulated genes displayed in two directions (Up and Down) were defined by positive and negative Spearman’s rho. The horizontal dotted lines represent *p*-value = 0.01 (Spearman’s rho test). Genes observed in both *w*- and *e*-GMS at *p*-value < 0.01 (Spearman’s rho test) and FDR < 0.25 (Benjamini & Hochberg correction) are black circled and labeled with gene name/id in the *e-*GMS plot. The relationships between parasite clearance time (pink blocks), gene expression (blue blocks) and SNP markers (yellow blocks) in *e-*GMS are characterized into five putative causality modes. **c** SNP affects clearance time dependent on expression and vice versa. **d** Clearance time and expression independently affects same SNP(s). **e** SNP affects clearance time independent of expression. **f** Expression mediated genetic effects on clearance time. **g** Expression affects clearance time independently of SNPs. Blocks are linked at *p*-value < 1e−05 for SNP-expression (eQTL) and SNP-clearance time (GWAS), and at *p*-value < 0.01 and FDR < 0.25 for expression-clearance time (TWAS). Corresponding gene/SNP name(s)/ID(s) or numbers are listed above blocks accordingly for each mode. Examples of resistance-associated relationships are shown in scatterplots with genes of farnesyltransferase (PF3D7_1242600) and thioredoxin-like (PF3D7_0717699) for mode **c**, 5′–3′ exonuclease (PF3D7_0204600) for mode **d**, 6-cysteine (PF3D7_0404900) and MDR1 (PF3D7_0523000) for mode **f**. Light blue used in scatterplots stand for isolates with reference alleles (ref.) and magenta for the alternative (alt). Expression is compared between alleles in boxplot on the right for each gene with the eQTL *p*-values labeled above

Taking the regional differences into consideration, we performed a genome- and transcriptome-wide association study (G/TWAS) of artemisinin resistance with the 229 *w*-GMS and 544 *e*-GMS isolates separately (Fig. 4). The clinical phenotype of artemisinin resistance was expressed as the parasite clearance time^5,18,31,32^. GWAS in the *e*-GMS confirmed a strong association with the *pfk13* C580Y and three previously identified SNPs^18^ producing a N96D substitution in gene PF3D7_1343100 of unknown function, N821K in *pfrad5* (DNA repair protein PF3D7_1343400)^32^ and a synonymous substitution of 103L in *pfap2-o4* (ApiAP2 transcription factor PF3D7_1350900), but also a previously unidentified intergenic SNP between gene PF3D7_1412400 and *actin* II (PF3D7_1412500) (Fig. 4a and Supplementary Table 2). In the *w*-GMS, GWAS identified two polymorphisms including a synonymous SNP of 501 T in gene PF3D7_0827600 and a nonsynonymous SNP, H511R, in *pfripr* (PF3D7_0323400) encoding for a Rh5-interacting protein involved in host cell invasion (Supplementary Table 3). In contrast, the TWAS identified as many as 365 genes whose transcriptional levels were associated with artemisinin resistance in either or both the *e*-GMS (Supplementary Data 10) and *w*-GMS (Supplementary Data 11) with equal statistical significance cutoff of *p*-value < 0.01 (Spearman’s rho test) and FDR < 0.25 (Benjamini & Hochberg correction) (Fig. 4b). Crucially, the majority of the artemisinin resistance-linked transcripts differed between *e*-GMS and w-GMS with only 19 genes upregulated and four genes downregulated commonly between the two GMS divisions at FDR < 0.25 (Fig. 4b, black circled). These included *pfmdr1*, *pfap2-tel*, *pfap2-sp*, *pfprp2,* and *pfmsp7*-like. However, in spite of the major differences there was a general agreement in the functional assignments of differentially expressed genes involved in artemisinin resistance, which include factors of ribosome biogenesis, RNA metabolism and exported proteins (Supplementary Fig. 7). Similarly, autophagy, DNA replication, cell adhesion, cell redox homeostasis and ribosomal structure exhibited down-regulation in both divisions. Interestingly, there was also common upregulation for several global cellular processes; albeit different sub-components detected in either of the division. The main example is the UPR, a putative effector of artemisinin resistance^20^, with factors related to ER stress upregulated in *e*-GMS and ubiquitin-related processes and proteasome-mediated degradation upregulated in *w*-GMS. Finally, we also observed distinct regional differences in artemisinin resistance-associated upregulation including genes involved in models for ubiquitin chain amputation in *e*-GMS and intracellular protein transport in *w*-GMS (Supplementary Data 12**)**. Overall, the TWAS showed a stronger correlation of transcription levels to artemisinin resistance in *e*-GMS with 275 genes compared to 113 genes in the *w*-GMS (*p*-value < 0.01 and FDR < 0.25).

To better understand the causality of artemisinin resistance, we investigated further the results from GWAS, TWAS and eQTLs. We found that two out of the five SNP markers identified by GWAS, the *pfk13* C580Y and a synonymous SNP in *pfap2-o4*, were linked with transcription in *e*-GMS (Fig. 4c). In particular, *pfk13* C580Y was positively correlated with the expression of farnesyltransferase (PF3D7_1242600) at *p*-value = 9e−06 (FDR = 0.006, permutation test) and negatively correlated with that of thioredoxin-like protein (PF3D7_0717900) at *p*-value = 2e−06 (FDR = 0.03, permutation test). Transcription of genes coding for farnesyltransferase and thioredoxin-like protein were also related to artemisinin resistance at *p*-value = 1e−05 (FDR = 0.006, permutation test) and *p*-value = 2e−04 (FDR = 0.03, permutation test), respectively (Fig. 4c). On the other hand, the putative interaction between *pfap2-o4* and 5′–3′ exonuclease (PF3D7_0204600) (eQTL *p*-value = 2e−06 and FDR = 0.02, permutation test) appears independent of artemisinin resistance (TWAS *p*-value = 0.4, Spearman’s rho test) (Fig. 4d). The other three SNP markers associated with artemisinin resistance in *e*-GMS showed no eQTLs-like effects on any *P. falciparum* gene (Fig. 4e). However, our eQTL analysis detected a set of 363 SNPs, which could be linked with artemisinin resistance via regulating expression of 141 genes (Fig. 4f and Supplementary Data 13). Two examples of such eQTL-expression pairs are a local linkage wiring PF3D7_0404800 C9Y (Pf3D7_04_v3-266145) to the 6-cysteine gene (PF3D7_0404900, *p*-value = 2e−07) and a distal linkage wiring PF3D7_1024400 P428A (Pf3D7_10_v3-1023588) to the MDR1 gene (PF3D7_0523000, *p*-value = 4e−09). It is feasible to suggest that at least some of the 363 eQTLs contribute to the genetic background of artemisinin-esistant parasites. These polymorphisms could operate together with another 132 genes whose expression is associated with resistance but are independent of genetic control (Fig. 4g). Similarly, *w*-GMS data defined another genetic background of 108 eQTLs linking to artemisinin resistance via controlling transcription variation of 47 genes (Supplementary Data 14).

## Discussion

Earlier studies on in vivo samples of *P. falciparum* have revealed that major variation of transcriptional levels occurs in genes encoding factors involved in host-parasite interactions^33,34^. This concept was subsequently expanded into the view of distinct physiological states of malaria parasites in vivo defined by transcriptional regulation of a broader spectrum of genes with multiple biological functions^35^. Specifically, it was proposed that the parasite cellular physiology could be (at least in part) controlled by fine-tuned transcriptional profiles of genes regulating active growth via glycolysis and/or alternative carbon source metabolism as well as other environmental stress responses. Distinct global transcriptional variations can also be associated with disease-related phenotypes such as malaria infection during pregnancy^36,37^ and severe malaria with cerebral complications^38,39^. While pregnancy-associated malaria seems mainly associated with upregulation of genes encoding specific surface antigens (such as *var* and *phist* genes), the severe infections involve parasites with more complex transcriptional changes in processes such as energy metabolism, biosynthesis, protein synthesis and folding, and cytoadhesion. Global transcriptional profiles seem to also govern distinct physiologies selected by transmission^40^. In particular, genes associated with fast growth and development such as transcription, translation, DNA replication, and energy metabolism are upregulated in parasites with high transmission while factors of sexual reproduction, motility and lipid metabolism are induced in parasites in low transmission areas. Following our initial work^20^, here we show that distinct transcriptional/physiological states also occur amongst parasite populations with similar transmission levels and in patients with similar disease outcome (uncomplicated malaria in semi-immune individuals)^5^. These results suggest that transcriptional regulation underlies parasite adaptation on even a finer scale reflecting local geographical regions potentially driven by subtle variations in malaria epidemiological aspects. These reflect the evolution of genetically distinct *P. falciparum* sub-populations in Western Cambodia that are thought to occur due to several factors, such as inbreeding, low transmission rate, geographical and/or reproductive isolation, high drug pressure and oxidative stress (due to hemoglobinopathies)^31^.

This eQTL analysis of *P. falciparum* in vivo isolates mirrors the previous in vitro study of the progeny of a genetic cross between two *P. falciparum* strains. Both studies showed that the distal eQTLs are more common compared to the local eQTLs, and that many of these are clustered within genetic hotspots^41^. Similarly, a large portion of these in vitro linkages involved a broad spectrum of protein functions beyond transcriptional regulation, with sequence polymorphisms altering their biological properties (e.g., missense mutations) and/or expression (e.g., CNV)^42,43^. These profiles of *P. falciparum* eQTLs from both in vitro samples^41^ and in vivo samples (shown here) are similar to those observed in other unicellular organisms such as yeast^24^. In *Saccharomyces cerevisiae*, the majority of transcriptional variations can be linked to *cis*- or *trans*-acting distal genetic loci involving factors of signal transduction, cytoskeleton, and protein metabolism as well as enzymes and proteins of other basic cellular function appeared to regulate transcriptional diversity^44,45^. Here, we demonstrated that malaria parasites also possess such a regulatory network that allows them to acquire differential physiological states and thus adapt to their unique host environment. The distal (*cis*- and *trans*-acting) linkages appear to facilitate these long-term adaptations, likely reflecting unique epidemiological factors that differentiate parasite populations in large geographical regions such as *e*-GMS and *w*-GMS. This regional adaptation likely results from a long-term accumulation of subtle variations of distal eQTLs involving a broad spectrum of cellular functions ranging from growth and development to basic metabolism and host cell remodeling^40^. However, besides the genetically driven transcriptional changes, some of the detected transcriptional variations are undoubtedly also controlled epigenetically. This may involve factors of heterochromatin, such as *P. falciparum* heterochromatin protein (PfHP1) that was shown to associate predominantly with transcriptionally variable genes^46^; and the histone 4 acetylation at lysine 8 (H4K8ac) marker that was shown to regulate several factors of growth and host-parasite interaction in the reverse relationship^47^. In summary, notwithstanding the epigenetic effect, it is feasible to speculate that the distal eQTLs may represent key components of putative genetic background(s) that maintain specific transcriptional and physiological states, which could predispose the malaria parasites to major phenotypic shifts, such as drug resistance^18^.

Here, we explored this possibility by investigating artemisinin resistance as the major phenotypic transformation that is currently ongoing in the GMS^5,16,48^. Previously, we have shown that artemisinin resistance (mediated by PfK13 mutations) is more likely to occur in parasites with upregulated UPR pathways and decelerated IDC progression^20,21^. Now, we extend this model with several additional observations. Firstly, while in most sites of the GMS the parasite cohorts exhibit a strong physiological conversion, in Pailin and Pursat, the epicenters of artemisinin resistance^4^, the parasites are transcriptionally highly diverse (Fig. 1b). Such physiological flexibility can boost the likelihood of initial emergence and local spread of drug resistance by selecting additional supporting genetic changes. This is consistent with the observations that PfK13 mutations were initially rising in multiple events along the GMS^15^ but were ultimately overrun by a dominating haplotype with C580Y SNP spreading from Western Cambodia^3,16^. In the future, it will be crucial to decipher the full catalog of these putative supportive variations that renders the Pailin genotypes most successful. This information will be particularly crucial to understand and monitor the spread of drug resistance beyond the GMS^49^ and ultimately to Africa where currently PfK13 mutations occur in very low frequencies and do not confer any resistant phenotype^50^. Second, we observed a major division in parasite physiology between the east and western part of the GMS that is mediated by essentially mutually exclusive sets of distal eQTLs (Fig. 3c) as well as differential frequencies of transcriptional upregulations (Fig. 2b). This further supports the initial genetic studies^18^, and indicates a potential of additional genetic/physiological backgrounds that promote the spread of artemisinin resistance based on distinct genotypes. These genotypes could include distinct PfK13 mutations such as F446I first reported in Myanmar^51^. Third, while the PfK13 polymorphisms remain currently the most predictive markers^52^, we identified a new set of genetic and transcriptional variations that significantly associate with artemisinin resistance (Fig. 4c–g). In particular, we identified up to 405 genes whose expression is related to artemisinin resistance and from these 167 genes are linked with 496 distal eQTLs. These factors may also contribute to artemisinin resistance either as direct mechanistic components or alleviating elements as a part of a complex genetic trait. It is feasible to suggest that these genetic/transcriptional factors are holding the key in a potential spread of PfK13-dependent resistance phenotypes (given the possibility of spontaneous rise of PfK13 SNPs) or constitute a new PfK13-independent mechanism(s), some of which were reported in vivo^53,54^ and in vitro^55^.

## Methods

### Ethics statement

In this study, we analyzed published data of *P. falciparum* parasites including SNPs and transcriptome which were derived from field samples. All studies providing the data were approved by the appropriate local ethics committees or the Oxford Tropical Research Ethics Committee. All samples were collected from patients’ blood with informed consent from the patient or a parent or guardian.

### Filtration of single nucleotide polymorphisms and samples

The SNP dataset was provided by the Sanger Institute. SNPs were discovered against the *P. falciparum* 3D7 reference sequence V3 using 2375 samples from Africa and Asia. It is accessible via [ftp://ngs.sanger.ac.uk/production/pf3k/release_5/5.1/]. We extracted out 2,174,070 high quality polymorphisms for 1069 isolate samples which had their transcriptome measured. To reduce artifacts and effects of complex infections, we only selected SNPs with two alleles and the minimal allele frequency (MAF) > 0.01 across the GMS. We also discarded samples with more than half of all SNPs not established or presenting other than biallelic polymorphisms. Finally, we considered 28,594 high quality SNPs and 773 isolate samples in this study.

### *P. falciparum* transcriptome

The raw data of 773 *P. falciparum* transcriptomes in this study was identical to that in Mok’s study^20^ which are accessible through NCBI’s Gene Expression Omnibus (GEO) Series accession number GSE59099. The data was processed by loess-normalization within arrays followed by quantile-normalization between samples/arrays using the Limma^56^ package of R. This method identified a transcriptome of 5061 genes which were represented in >67% of all samples/arrays with probes showing median foreground intensity > 1.5-fold median background intensity for either channel. Missing values (<2% of total detected log2 ratios) were refilled by nearest neighbor averaging algorithm in the R package of impute. The processed expression data of 773 isolates are provided in Supplementary Data 15.

### Estimation of parasites HPI and GAM proportion

For each individual parasite population/sample, we estimated its most proximate age (hours post invasion, HPI) during the asexual intraerythrocytic developmental cycle (IDC) and the proportion of gametocytes using a mixture model based on a maximum likelihood method. The method is adjusted from the model described in Lemieux’s work^57^. In this study, the mixture model is constructed by some proportion of asexual parasites and another proportion of gametocytes that is presented in formula as:

1 $$y_{\mathrm{g}} = \left( {1 - \alpha } \right)^\ast x_{\mathrm{g}}\left( {{\mathrm{hr}}} \right) + \alpha ^\ast z_{\mathrm{g}}\left( d \right) + \varepsilon _{\mathrm{g}}$$

where *y*_g_ is the gene expression values of a field site sample; *α* is the proportion of gametocytes to the total parasite count (sum of gametocytes and asexual parasites); *x*_g_(hr) is the gene expression values of the reference asexual sample at the HPI of hour hr; *z*_g_ is the gene expression values of the reference gametocyte sample at the day of *d* and the *ε*_g_ is the associated error term. The error term *ε*_g_ is estimated using differences between all the isolate samples and lab strains. The reference transcriptomes of asexual stages were generated by Bernardo et al.^22^ in the *P. falciparum* Dd2 strain with 24 time points obtained during the IDC with 2 h intervals. To estimate HPI at a high resolution, the reference IDC transcriptomes were interpolated into 240 time points with 0.2 h intervals using smooth splines in this study. The reference gametocyte transcriptome were generated in 3D7 strain parasites after PfHP1 depletion^58^ and collected at the 1st to 12th day during the parasites gametocyte development. The data was uploaded to Gene Expression Omnibus and the accession number is GSE121505. The R script is provided in Supplementary Software 1.

The principal component analysis (PCA) on 32 reference transcriptomes (24 from asexual parasites and eight from gametocyte parasites) reveals that a separation of IDC and GAM transcriptomes is clearly interpretable by the first three PCs (Supplementary Fig. 1f, panel on the top right). Based on the top three PCs, we observed strong differences among field site isolates in their HPI age of asexual parasites and proportions of gametocytes by projecting TRACI samples onto the space of the first three PCs (Supplementary Fig. 1e, f).

### HPI and GAM adjustment

In order to remove the expression variation caused by parasite age of an individual sample and its proportion of GAM parasites, we corrected expression values for each gene by extracting expression residuals from the fitted linear model where HPI and GAM proportion were used as covariates. To fit the linear model, we utilized the logit transformation of GAM proportion and also added a covariate of squared HPI due to the polynomial-like relationship between the expression profiles and parasite age. PCA to the adjusted data showed a successful reduction of impact of GAM and HPI on the transcriptome (Supplementary Fig. 1c, d). The adjusted expression data is provided in Supplementary Data 16.

### Transcriptome Local Conservation index (*Tcvs*)

*Tcvs* score was designed to measure the distance of an individual transcriptome to the local cohort against the entire cohort of whole GMS. The *Tcvs* is defined as:

2 $$Tcvs_i = 1 - \frac{{{\mathrm{ED}}_{{\mathrm{Ti.within}}}}}{{{\mathrm{ED}}_{{\mathrm{Ti.broad}}}}}$$

where ED_Ti.within_ represents the average Euclidean distance of transcriptomes between the *i*th sample to randomly selected 1000 samples from its local field site; ED_Ti.broad_ represents the average Euclidean distance of transcriptomes between the *i*th sample to randomly selected 1000 samples from the entire GMS pool. The transcriptome pool of GMS parasites was generated by 5000 times sub-sampling with each field site cohorts presented at the same frequency. The Euclidean distance of two transcriptomes was calculated based on the standardized expression data after HPI and GAM proportion adjustment.

To investigate functional gene sets associated to the local convergence/divergence of parasites transcriptomes across the GMS, we developed a gene expression fixation index (*geFST*) to measure the expression differentiation at each field site for individual genes. The *geFST* is defined as:

3 $$geFST_{gi} = 1 - \frac{{{\mathrm{ED}}_{{\mathrm{gi.within}}}}}{{{\mathrm{ED}}_{{\mathrm{gi.broad}}}}}$$

The Euclidean distance was calculated for the *i*th gene, *gi*, between two randomly selected isolates. Here, ED_gi.within_ represents the average Euclidean distance of 1000 random isolate pairs sampled within a local field site; and ED_gi.broad_ represents the average Euclidean distance between 1000 random isolate pairs sampled from the given field site and entire GMS pool. Then, *geFST* values were normalized into *z*-scores for each field site and the averaged *z*-scores were calculated for 5061 individual genes across field sites and used in the following GSEA study. The most conserved/diverged pathways throughout all field sites were determined in GSEA at *p*-value < 0.05 and FDR < 0.25 based on isolates presenting positive *Tcvs* scores. For the field sites of Pursat and Pailin, which displayed the highest transcriptome diversion (see main text), *geFST* was individually calculated using the entire set of local samples, 89 isolates at Pursat and 76 at Pailin. It resulted in 106 and 147 significantly diverged gene expressions at *z*-score < −2 for Pursat and Pailin, respectively. The pathways enriched of diverged genes at Pursat/Pailin were obtained at *p*-value < 0.05 with hypergeometric test. Full list of enriched pathways are listed in Supplementary Data 3.

To compare parasites transcriptome relatedness to the genome, we calculated the Genome Local Conservation index (*Gcvs*) analogous to the *T*cvs index. The *Gcvs* is defined as:

4 $$Gcvs_i = 1 - \frac{{D_{{\mathrm{Gi.within}}}}}{{D_{{\mathrm{Gi.broad}}}}}$$

where *D*_*Gi*.within_ represents the average genetic distance between the *i*th sample to randomly selected 1000 samples from its local field site. *D*_*Gi*.broad_ represents the average genetic distance between the *i*th sample to randomly selected 1000 samples from the entire GMS pool. The pairwise genetic distance of samples was estimated based on the genetic similarity matrix which was generated by FaST-LMM using unlinked SNPs. We subtracted the similarity scores from 1 and used that to indicate the genetic distance between samples.

### Transcriptome population structure

The transcriptome population structure was reconstructed using a neighbor-joining tree for the 773 isolate samples across eleven field sites of the GMS. The distance matrix used for clustering was calculated as Euclidean distance of pairwise transcriptomes based on the corrected expression data (HPI and GAM proportion adjusted), which was also centered to the sample mean with the standard deviation scaled to 1.

### Population stratification

Samples within the *e*/*w*-GMS still present subpopulation stratifications. The possible population structure was estimated using four multidimensional scaling (MDS) vectors, which was obtained from the 16,614 unlinked SNPs. The 16,614 unlinked SNPs were defined out of the 28,594 SNPs using Plink v2.8 LD-based variant pruner with –indep-pairwise option, parameter settings of window size=100 kb, and the square correlation cutoff *R*^2^ > 0.3 in step size of 10.

### eQTL mapping

The eQTL mapping was performed for *e*-GMS and *w*-GMS independently using the same processing pipeline. For each region, the corrected expression data of 5061 genes were considered as 5061 traits and mapped to the 28,594 high quality SNPs. The test of SNP-expression association was implemented using FaST-LMM v2.07^59^ with the genetic similarity matrix estimated based on unlinked SNPs.

### Gene expression correction and PCA adjustment

To achieve more detectable eQTLs we processed expression data with the following procedures aiming to minimize the impact of non-genetic components on the expression variations, which is generally applied to eQTL analyses in other systems also^24–28^. First, utilizing the four MDS vectors estimated on the above, the expression data was corrected for the possible population structure by extracting the residuals from a linear regression. Second, a PCA was performed to the 544 *e*-GMS (229 *w*-GMS) transcriptomes to generate top 50 principal components (PCs) to capture major expression differences among samples which may be due to physiological, environmental or systematic experimental variations^24–28^. Association analysis of those PCs confirmed their correlations to the estimated parasite age (HPI), gametocyte proportion, and numbers of known clinical factors like parasite counts, sample origin, patient temperature and other host-related factors (Supplementary Data 1). Third, we picked top 20 PCs for expression correction based on the inflection point of the percent variation curve of the 50 PCs (Supplementary Fig. 8). In addition, the eQTL numbers on chromosome 13 were also optimized at 20 PCs correction with no significant increment by removing more. Fourth, we performed QTL analysis for the 20 PCs by mapping each to the 28,594 SNPs using FaST-LMM. According to their relationships to genotype, we subtracted 5 of the 20 PCs from the next correction steps for *e*-GMS. Finally, expression values were corrected for each gene by determining the residuals using linear regression with the selected PCs as covariates. For the *w*-GMS dataset, we applied the same procedure with considering only top 10 PCs because of the smaller sample size of 229 isolates.

After expression adjustment, PCA was repeated to the adjusted data to investigate the efficiency of removing non-genetic component effects. The results revealed a successful reduction of environmental effects from the adjusted expression values (Supplementary Data 1). All the potential environmental factors, the known and estimated, are listed in Supplementary Data 17.

### eQTLs quality control

Since eQTL mapping generates multiple testing results and the confounding effects vary across genes and may hence not be fully captured by the top 20 PCA components, we applied expression permutation to control the FDR for the individual populations (*w*-GMS and *e*-GMS parasites) independently. For each gene, we shuffled the sample IDs within sites to maintain the transcriptome structure of sub-region isolates. Given the heavy computation of permutations required by eQTLs analysis, the permutation was performed in a small scale for 2400 genes which was randomly selected from the potential targets of eQTLs (*p*-value < 1e−05); and the mapping was restricted to unlinked SNPs derived from Plink LD pruning (-indep-pairwise 1000 20 0.2 -maf 0.01) which resulted in 9229 SNPs for *e*-GMS and 8422 SNPs for *w*-GMS. Next, according to the permutation results, we constructed the null distribution of p-values for eQTL mappings to SNPs with 0.01 < MAF < 0.05 separately from those to SNPs with MAF > 0.05, aiming to provide more justified FDR values to SNP-expression associations with different MAF levels. The results showed that the cutoff of p-value < 1e−5 called *e*-GMS eQTLs at FDR < 0.06 for SNPs with MAF > 0.05, and FDR < 0.2 for SNPs with 0.01 < MAF < 0.05; and called *w*-GMS eQTLs correspondingly at FDR < 0.1 and FDR < 0.25 for each MAF category. The estimated FDR values are provided together with p-values for each eQTL in Supplementary Data 5 and 6.

With the cutoff of p-value < 1e−05, the eQTL mapping initially detected 9,541 SNP-expression linkages for *e*-GMS and 11,293 linkages for *w*-GMS with the input data of 5061 genes expression and 28,594 high quality SNPs. For each detected linkage of SNP-expression pair, we further performed 100 times permutation tests with shuffling the sample IDs within sites per time. A linkage would be discarded if any permutation results passed the threshold of *p*-value < 1e−5. This removed 820 linkages from *e*-GMS and 6756 linkages from *w*-GMS. Finally, the eQTL mapping resulted in 8720 SNP-expression linkages in *e*-GMS and 4537 linkages in *w*-GMS.

To achieve a high-confidence dataset for holistic eQTL analysis in *P. falciparum* parasites, we pruned the union results from *e*-GMS and *w*-GMS by removing linkages showing contradictory association effects between them. In practice, we ignored linkages with positive log2 odds ratio (LOD) in one sub-region but negative LOD in the other; if a SNP with very low minimum allele counts (≤5) was found with low LOD (between −1 and 1), it was also removed from further analysis. Finally, the high-confidence dataset containing a total of 5575 SNP-expression linkages were composed of 3452 *e*-GMS linkages and 2170 *w*-GMS linkages. The 5575 linkages uncovered 3972 eQTL SNPs associating to transcriptional variations of 2350 genes. For the 3972 identified eQTL SNPs, 1072 (27%) underlay the eQTL regions observed by Gonzales’s in vitro study^41^.

### eQTL clusters

There were 2837 out of 3972 eQTLs falling beyond the linkage disequilibrium according to the Plink LD-based variant pruning described above. The eQTL clusters were defined based on the 2837 unlinked eQTLs. A 50 kb sliding window was used to scan regions along each chromosome. Windows with over-representation of eQTLs were defined at *p*-value < 0.05 by χ^2^ test against the expected number for each chromosome. Consecutive enriched windows were merged into one if the adjusted *p*-value < 0.05 was obtained for the merged window. Finally, it resulted in 58 eQTL clusters covering 47% (or 1867) of the 3972 eQTLs.

### Sub-sampling of eQTL mapping

Sub-samplings were performed to the *e*-GMS populations three times. Each round, 229 (the same number of *w*-GMS isolates) samples were randomly selected from the *e*-GMS populations and the eQTL mapping pipeline were repeated using all the same parameters previously applied to the *w*-GMS data analysis. The detail steps included re-estimating the population structures of sub-sampled isolates using multidimensional scaling factors, re-calculating PCA components of the transcriptome populations and the number of top components used for expression normalization. The SNP-expression association was tested for all the high-confidence 5575 SNP-expression linkages.

### Genome-wide association study (GWAS)

GWAS was performed using a linear mixed model in FaST-LMM v2.07 together with the correction for population structure. With the threshold of MAF > 0.01, we tested 24,652 SNPs for their associations to parasite clearance time in the *e*-GMS, and 22,216 SNPs in the *w*-GMS. The parasite clearance time denotes the parasite clearance half-lives estimated using a parasite clearance estimator^32,60^. The number of SNP markers detected at a gradual significant level are summarized in Supplementary Fig. 9a. A minor overlapping was observed between *w*-GMS and *e*-GMS. The full results of GWAS are listed in Supplementary Table 2 and 3.

### Transcriptome-wide association study (TWAS)

TWAS was carried out for the *e*-GMS and *w*-GMS populations individually by applying linear regressions between mRNA levels and parasite clearance time for each gene. The mRNA levels were corrected for HPI and GAM proportions before TWAS. In addition, due to the significant correlation between parasite clearance time and parasite origins, we removed the geographic effects from both of the variations of parasite clearance half-life and gene expression using the same method of residuals as described above. Spearman’s rho was used to test for association between expression and clearance time without samples, which showed outlier expression (*z*-score > 3, *z*-scores are differences between each value and the mean divided by standard deviation). The FDR was estimated using Benjamini & Hochberg correction on *p*-value. The number of gene markers detected at a gradual significant level are summarized in Supplementary Fig. 9b. Given the different sample size in *e*-GMS and *w*-GMS, we conducted 100 times sub-sampling to the *e*-GMS data by randomly selecting 229 samples each time. The Spearman’s rho estimated at 75% confidence interval from sub-sampling did not show much difference from the original ones. This reveals the strong correlation observed in TWAS of *e*-GMS than *w*-GMS was not due to the bigger sample size. The full results of TWAS are listed in Supplementary Data 10 and 11.

### Code availability

The R script of mixture model used for estimating parasites HPI and GAM is available in Supplementary Software 1. All the tools and softwares applied in this study are described within the methods.

## Electronic supplementary material

Supplementary Information

Description of Additional Supplementary Files

Supplementary Software 1

Supplementary Data 1

Supplementary Data 2

Supplementary Data 3

Supplementary Data 4

Supplementary_ Data 5

Supplementary Data 6

Supplementary Data 7

Supplementary Data 8

Supplementary Data 9

Supplementary Data 10

Supplementary_ Data 11

Supplementary Data 12

Supplementary Data 13

Supplementary Data 14

Supplementary Data 15

Supplementary Data 16

Supplementary Data 17

## Data Availability

All data supporting the findings of this study are available within the article and its Supplementary Information files, or from the corresponding author upon request. The reference gametocyte transcriptome has been uploaded to Gene Expression Omnibus under accession number GSE121505. The SNP dataset was provided by the Sanger Institute and is accessible via [ftp://ngs.sanger.ac.uk/production/pf3k/release_5/5.1/]. The raw data of *P. falciparum* transcriptomes is accessible through Gene Expression Omnibus (GEO) Series accession number GSE59099.
