# Supplementary material for: The origins of malaria artemisinin resistance defined by a genetic and transcriptomic background
Source: Nat Commun. 2018 Dec 4;9:5158. doi: 10.1038/s41467-018-07588-x (PMC6279830; doi:10.1038/s41467-018-07588-x)
Supplement: Supplementary file 1 — Supplementary Information [file 41467_2018_7588_MOESM1_ESM.pdf]

Supplementary Information

The origins of malaria artemisinin resistance defined by a genetic and transcriptomic background

Zhu et al.

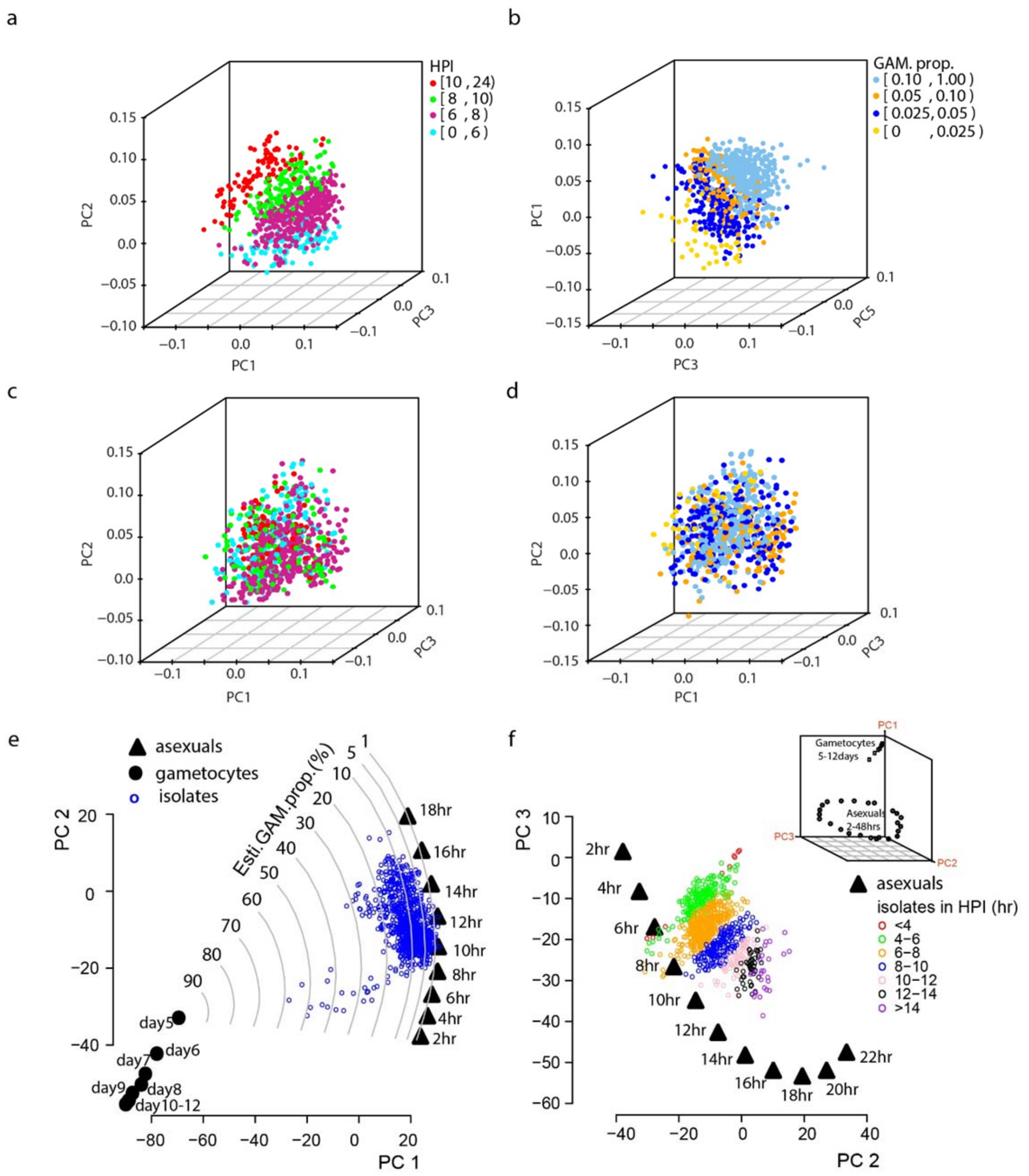
a
b
c
d
e
f
HPI
[10 , 24)
[8 , 10)
[6 , 8)
[0 , 6)
GAM. prop.
[0.10 , 1.00)
[0.05 , 0.10)
[0.025, 0.05)
[0 , 0.025)
PC1
PC2
PC3
PC5
asexuals
gametocytes
isolates
Esti.GAM.prop.(%)
PC 1
PC 2
PC 3
day5
day6
day7
day8
day9
day10-12
2hr
4hr
6hr
8hr
10hr
12hr
14hr
16hr
18hr
20hr
22hr
Gametocytes 5-12days
Asexuals 2-48hrs
isolates in HPI (hr)
<4
4–6
6–8
8–10
10–12
12–14
>14

Supplementary Fig. 1 PCA of *P. falciparum* transcriptome in the GMS. PCA of 773 isolates transcriptome reveals that (**a)** the top 3 PCs strongly reflect the dominant age (in hours post invasion, HPI) of asexual parasites during IDC progression; and (**b**) the first, third and fifth PCs are most correlated to the proportion of gametocytes (GAM prop.). The 773 isolates were stratified into four groups by their estimated HPI in 0-6 hr (light blue), 6-8 hr (purple), 8-10 hr (green) and >10 hr (red); and another four groups according to their estimated GAM prop. in 0-2.5% (yellow), 2.5-5% (blue), 5-10% (orange) and >10% (skyblue). The estimation of HPI and GAM was based on a mixture model described in Methods. After the HPI and GAM correction, PCA was repeated for the adjusted transcriptome of 773 isolates. HPI and GAM effects were successfully removed from the expression data which can be reflected by the scattered isolates plotted using top 3 new PCs for (**c**) HPI and (**d**) GAM with the same stratification groups in (a) and (b). In addition, we also preformed PCA to the 32 reference transcriptome (24 asexual and 8 gametocytes samples). (e) The GAM variation across isolates can be visualized in the space of PC1 and PC2 which mainly reflect the transcriptome difference between asexual and GAM samples. (f) The HPI variation across isolates can be visualized by the PC2 and PC3 which mainly reflect the difference between parasites in different ages. The panel on the top right in (f) is the space constructed by top 3 PCs with all the reference transcriptome projected onto it.

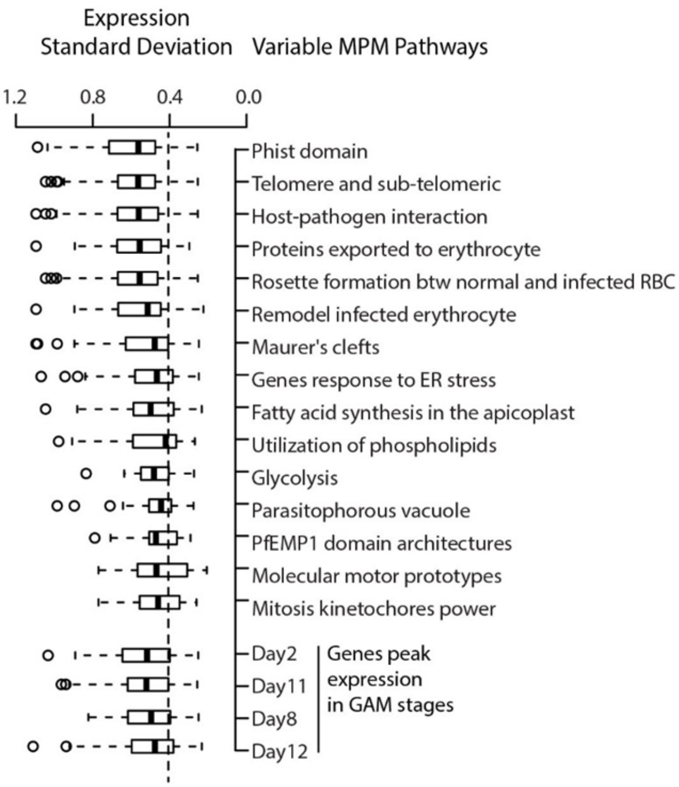

Supplementary Fig. 2 MPM pathways with high expression variability defined by GSEA at p-value<0.05 and FDR<0.25. The boxplot represents the standard deviation (SD) of adjusted expression values across 773 *P. falciparum* isolates for each gene group. The dot line represents the median SD of overall genes. The centre line, bounds and whiskers of each box represent the median, interquartile and 1.5 interquartile respectively.

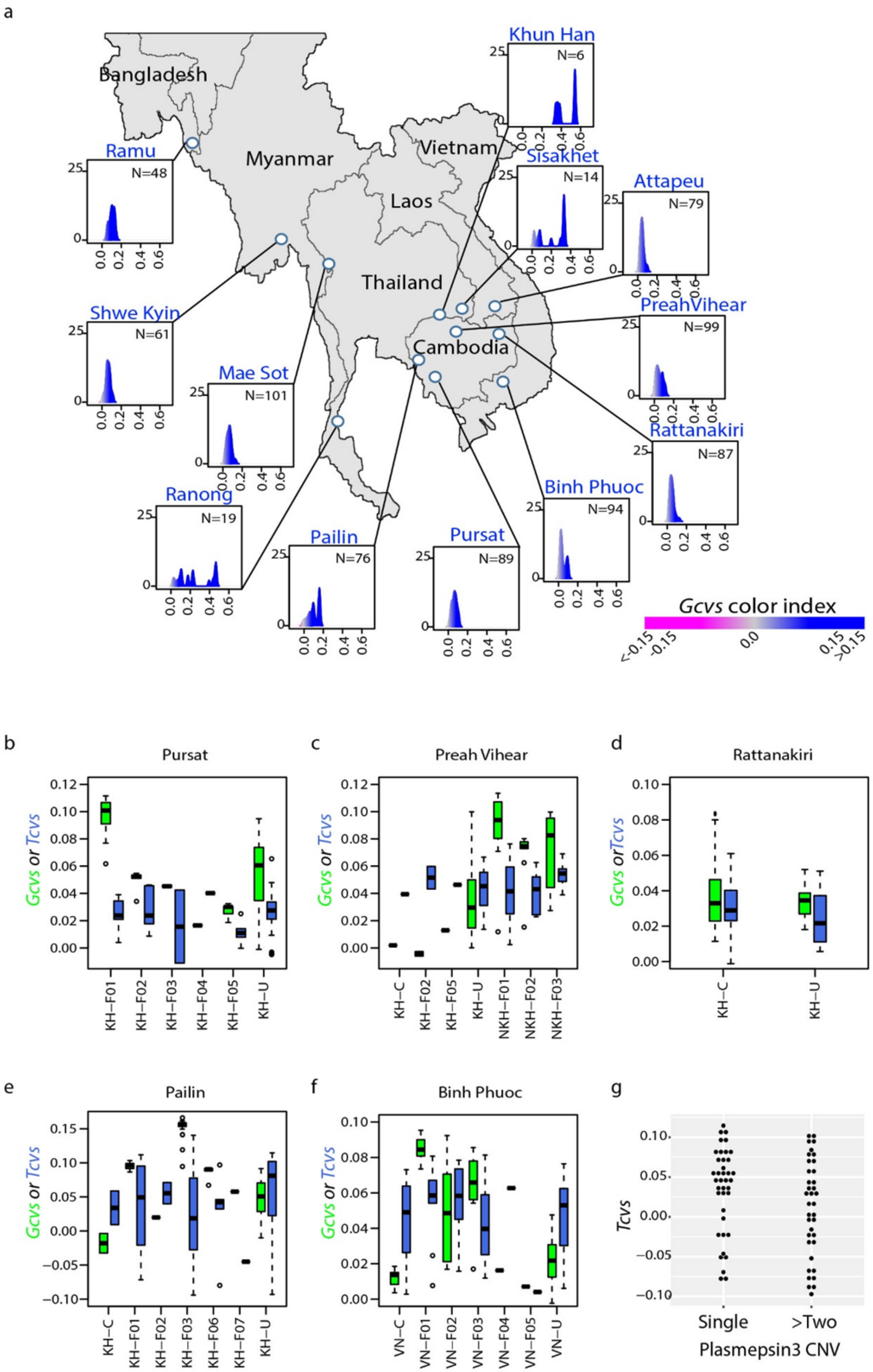

a
Bangladesh
Myanmar
Vietnam
Laos
Thailand
Cambodia
Khun Han N=6
Ramu N=48
Sisakhet N=14
Attapeu N=79
Shwe Kyin N=61
Mae Sot N=101
PreahVihear N=99
Rattanakiri N=87
Binh Phuoc N=94
Ranong N=19
Pailin N=76
Pursat N=89
Gcvs color index
<-0.15 -0.15 0.0 0.15 >0.15
b Pursat
c Preah Vihear
d Rattanakiri
e Pailin
f Binh Phuoc
g
Gcvs or Tcvs
KH−F01 KH−F02 KH−F03 KH−F04 KH−F05 KH−U
KH−C KH−F02 KH−F05 KH−U NKH−F01 NKH−F02 NKH−F03
KH−C KH−U
KH−C KH−F01 KH−F02 KH−F03 KH−F06 KH−F07 KH−U
VN−C VN−F01 VN−F02 VN−F03 VN−F04 VN−F05 VN−U
Tcvs
Single >Two
Plasmepsin3 CNV

Supplementary Fig. 3 A comparison of *P. falciparum* parasites genome and transcriptome relatedness in the GMS. (a) Distribution of *Gcvs* is displayed in density plot to show the genetic convergence of parasites at each TRACI-study sites of the 773 isolates. The geographic map of Southeast Asia is originally downloaded from https://freevectormaps.com/world-maps/southeast-asia and modified using Photoshop. The *Gcvs* values were analogously calculated like *Tcvs* based on genetic similarity matrix (Methods). Isolate numbers are shown together with the density plots for each site. Interestingly, the genetically defined founder-like (sub)population is distinguishable by the *Gcvs* distributions, but not the *Tcvs* distributions in some of the studied sites, e.g. (b) Pursat, (c) Preah Vihear, (d) Rattanakiri, (e) Pailin and (f) Binh Phuoc. (g) *Tcvs* are compared between isolates with single copy of plasmepsin 2/3 and those with multiple copies.

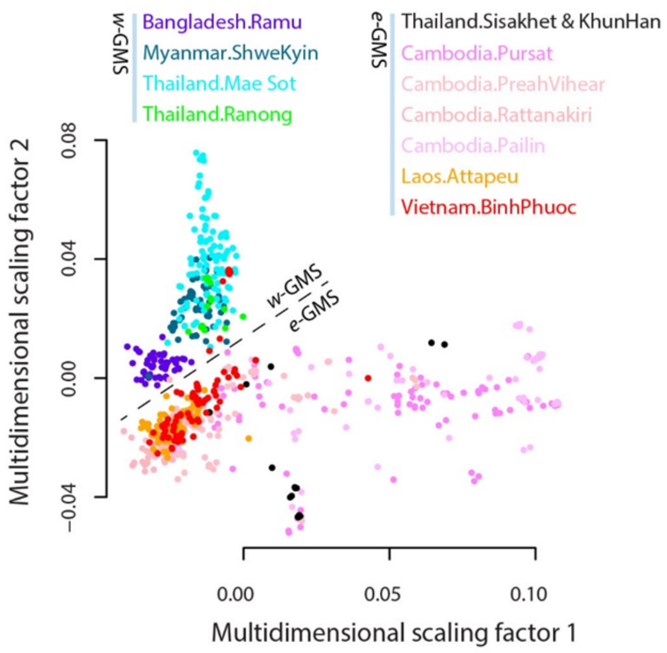

Supplementary Fig. 4 Multidimensional scaling analysis on 28,594 high quality SNPs represented by the first two factors (Methods). The black dotted line indicates the separation of isolates from *w*- and *e*-GMS.

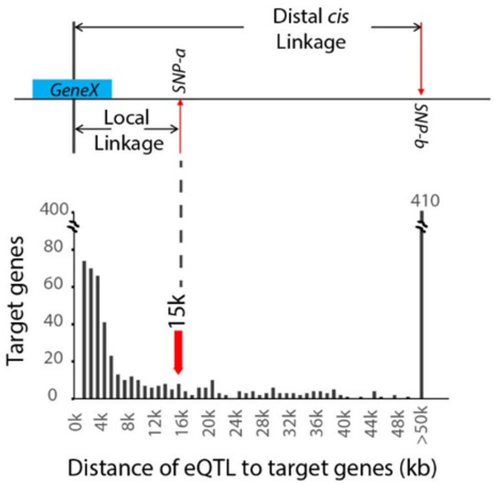

Supplementary Fig. 5 A schematic representation of local (<15 kb) and distal *cis* linkages (>15 kb). The bar graph represents the target genes in each category of linkages based on distance.

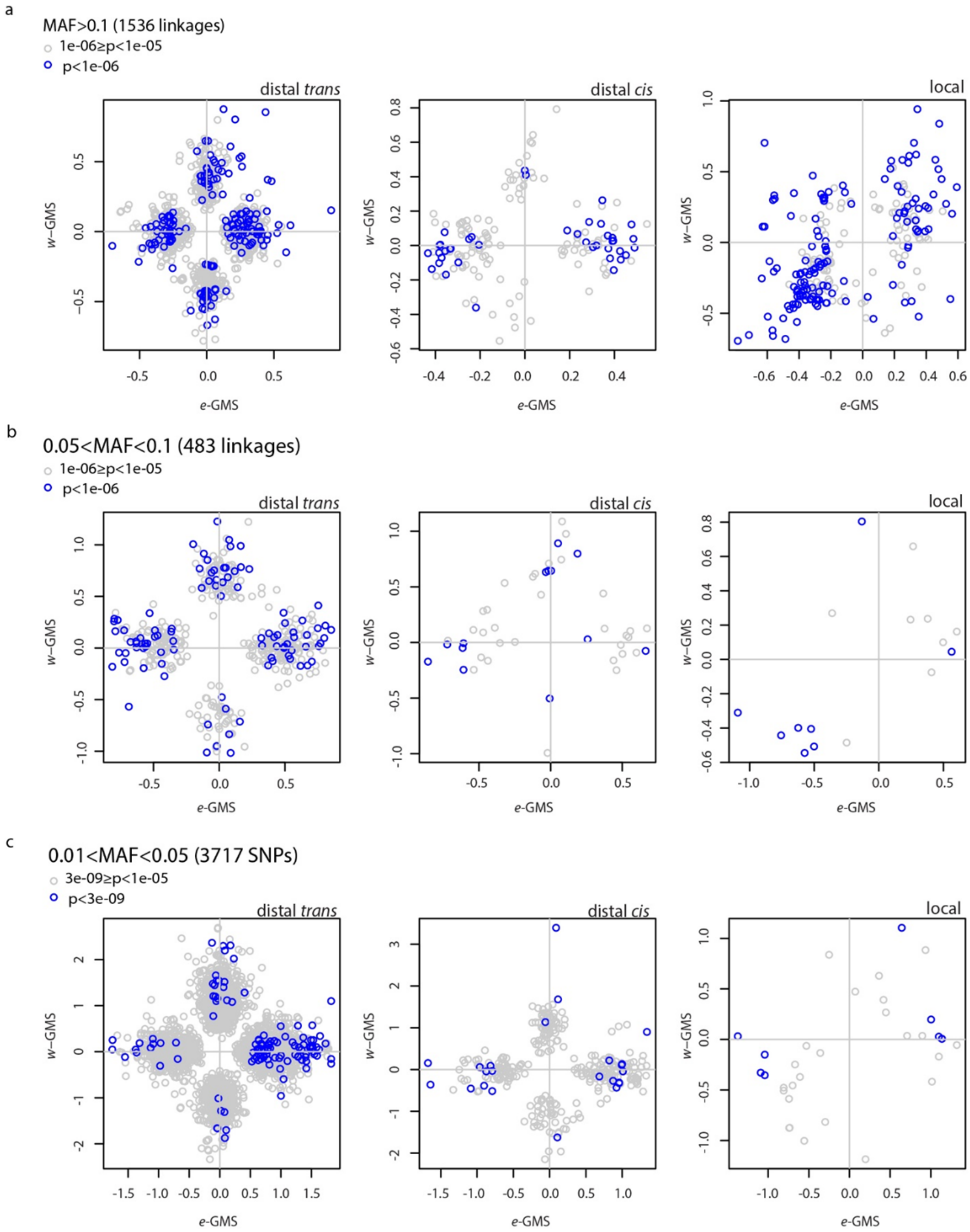
a
MAF>0.1 (1536 linkages)
1e-06≥p<1e-05
p<1e-06
distal trans
distal cis
local
w–GMS
e-GMS
b
0.05<MAF<0.1 (483 linkages)
1e-06≥p<1e-05
p<1e-06
distal trans
distal cis
local
w–GMS
e-GMS
c
0.01<MAF<0.05 (3717 SNPs)
3e-09≥p<1e-05
p<3e-09
distal trans
distal cis
local
w–GMS
e-GMS

Supplementary Fig. 6 Differential eQTL effects at different MAF level. LOD values are used to represent eQTL effects and plotted to compare eQTLs between *e*-GMS and *w*-GMS in three categories (*rows*) which are characterized by MAF levels. For each category, distal *trans* and *cis* interactions are displayed separately from the local interactions aiming to clearly visualize the impact of MAF and FDR settings on the observation of distinct eQTL pattern in (sub)regions (*e*- and *w*-GMS). (a) 1,536 eQTL linkages have SNPs with MAF>0.1 in both (sub)regions. Blue circles represent eQTLs passing $P<1e\text{-}6$ (FDR≤0.01); and grey circles for other eQTLs. (b) 483 linkages have SNPs with $0.05<MAF<0.1$ in both (sub)regions. Blue circles represent eQTLs passing $P<1e\text{-}6$ (FDR≤0.01); and grey circles for other eQTLs. (c) 3,717 linkages have SNPs with $0.01<MAF<0.05$ in both (sub)regions. Blue circles represent eQTLs passing $P<3e\text{-}9$ (FDR≤0.01); and grey circles for other eQTLs.

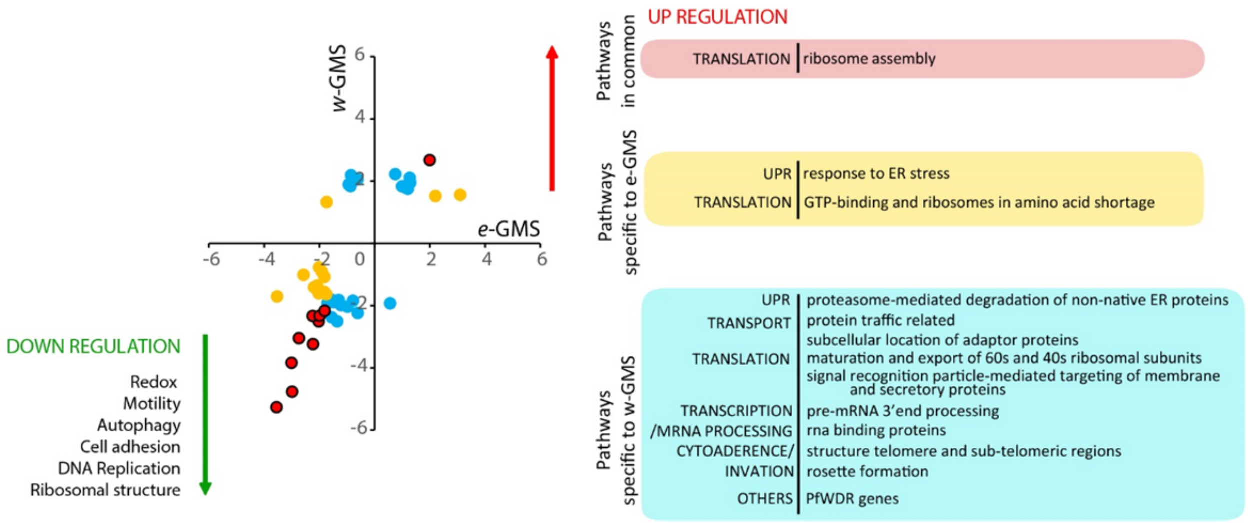

Supplementary Fig. 7 Functional enrichment analysis of TWAS by GSEA. GSEA was applied to the pre-ranked 5,061 genes based on their Spearman's *rho* of expression to parasite clearance time. Significant MPM pathways are defined at p-value<0.05 and FDR<0.25. Up/down regulations are defined by the positive/negative values of Normalized enrichment scores (NES). Finally, the NES are plotted between the *e*-GMS and *w*-GMS to show their functional agreement in artemisinin resistance-associated genes. Yellow dots stand for pathways derived from *e*-GMS, blue for those from *w*-GMS, and red for the common. Particular upregulated pathways are summarized on the right.

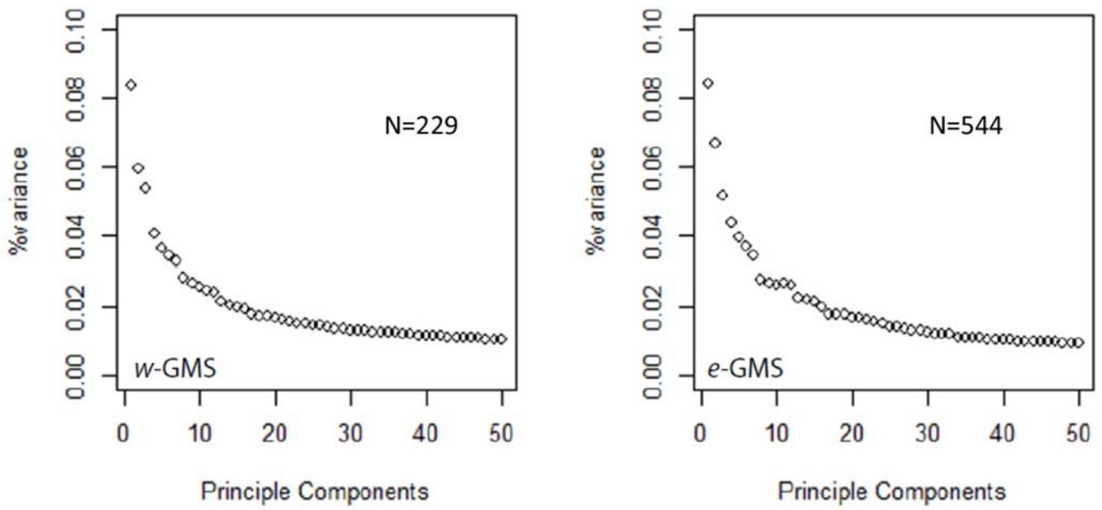

Supplementary Fig. 8 Percent variation curve for top 50 PCs for the *w*-GMS dataset (left) and the *e*-GMS dataset (right).

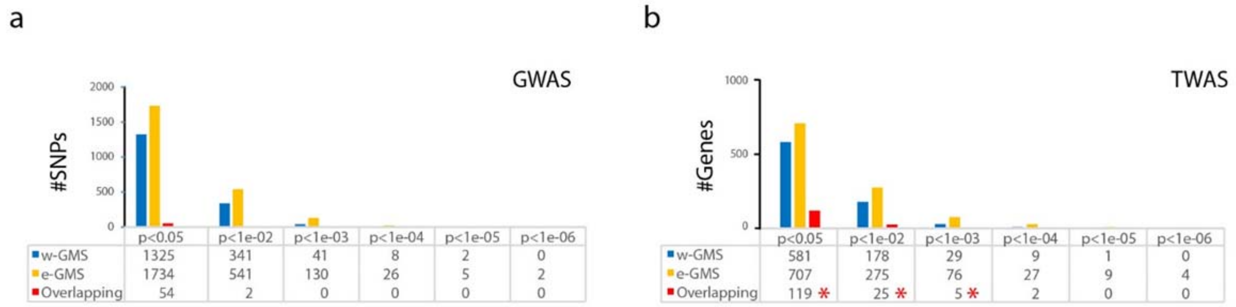

Supplementary Fig. 9 GWAS and TWAS results summary. **a** the bar plot are shown as resistance-associated SNPs detected at gradual p-value cutoffs for *w*-GMS(*blue*), *e*-GMS(*yellow*) and both (*red*). The corresponding numbers are listed in the table below. The *Overlapping* category describes SNPs showing concordant association effects (both positive/negative correlation) in the two (sub)regions. b the bar plot are shown as resistance-associated genes detected at gradual p-value cutoffs for *w*-GMS(*blue*), *e*-GMS(*yellow*) and both (*red*). The corresponding numbers are listed in the table below. The *Overlapping* category describes genes showing concordant association effects (both positive/negative correlation) in the two (sub)regions. Red asterisk marks the overrepresentation of common genes at $P<0.01$ (binomial test).

Supplementary Table 1 summary of SNP-expression linkages and eQTLs.

Linkage type	Linkage number	eQTL-regulated gene	eQTL				
			Total	Intergenic	Intron	Synonymous	Non-synonymous
Local	360	171	344	134	28	62	120
Distal *cis*	507	337	438	150	31	97	205
Distal *trans*	4708	2147	3381	1180	168	729	1304

Supplementary Table 2 Genome-wide association study (GWAS) of artemisinin resistance for *e*-GMS parasites (p-value<1e-4).

SNP	Chromosome	Position	Pvalue	Qvalue	OddsRatio
Pf3D7_13_v3-1725259	13	1725259	1.37E-18	3.38E-14	3.71
Pf3D7_13_v3-1717359	13	1717359	3.09E-08	3.81E-04	2.16
Pf3D7_13_v3-1700345	13	1700345	4.18E-06	3.43E-02	1.78
Pf3D7_14_v3-504379	14	504379	6.55E-06	3.88E-02	1.70
Pf3D7_13_v3-2028330	13	2028330	7.87E-06	3.88E-02	2.00
Pf3D7_14_v3-2481070	14	2481070	1.44E-05	5.36E-02	0.59
Pf3D7_13_v3-1947262	13	1947262	1.52E-05	5.36E-02	5.01
Pf3D7_13_v3-2114339	13	2114339	2.53E-05	6.40E-02	1.63
Pf3D7_14_v3-1956225	14	1956225	2.61E-05	6.40E-02	0.62
Pf3D7_11_v3-966435	11	966435	2.97E-05	6.40E-02	3.54
Pf3D7_12_v3-1681411	12	1681411	3.19E-05	6.40E-02	2.14
Pf3D7_14_v3-3036963	14	3036963	3.30E-05	6.40E-02	2.25
Pf3D7_13_v3-1867630	13	1867630	3.55E-05	6.40E-02	1.62
Pf3D7_14_v3-2568280	14	2568280	3.74E-05	6.40E-02	2.54
Pf3D7_04_v3-138376	4	138376	3.93E-05	6.40E-02	3.36
Pf3D7_12_v3-458770	12	458770	4.15E-05	6.40E-02	2.06
Pf3D7_08_v3-640323	8	640323	4.71E-05	6.83E-02	0.65
Pf3D7_13_v3-690489	13	690489	6.14E-05	7.91E-02	1.73
Pf3D7_13_v3-1898712	13	1898712	6.29E-05	7.91E-02	4.23
Pf3D7_08_v3-561418	8	561418	6.41E-05	7.91E-02	1.60
Pf3D7_13_v3-1831070	13	1831070	6.94E-05	8.14E-02	2.14
Pf3D7_14_v3-2513850	14	2513850	8.13E-05	8.68E-02	1.56
Pf3D7_14_v3-2492156	14	2492156	8.15E-05	8.68E-02	0.63
Pf3D7_04_v3-1100467	4	1100467	8.45E-05	8.68E-02	4.87
Pf3D7_06_v3-784632	6	784632	9.04E-05	8.77E-02	3.70
Pf3D7_12_v3-2119411	12	2119411	9.25E-05	8.77E-02	3.16

Supplementary Table 3 Genome-wide association study (GWAS) of artemisinin resistance for *w*-GMS parasites (p-value<1e-4).

SNP	Chromosome	Position	Pvalue	Qvalue	OddsRatio
Pf3D7_03_v3-982435	3	982435	3.50E-06	7.77E-02	2.77
Pf3D7_08_v3-1191426	8	1191426	9.64E-06	1.07E-01	8.50
Pf3D7_12_v3-2065087	12	2065087	2.04E-05	1.50E-01	5.13
Pf3D7_14_v3-35821	14	35821	2.70E-05	1.50E-01	3.25
Pf3D7_11_v3-332295	11	332295	4.23E-05	1.81E-01	2.33
Pf3D7_14_v3-84342	14	84342	4.88E-05	1.81E-01	2.36
Pf3D7_10_v3-1482930	10	1482930	8.88E-05	2.68E-01	3.26
Pf3D7_10_v3-1481769	10	1481769	9.65E-05	2.68E-01	3.24
