# Supplementary material for: The origins of malaria artemisinin resistance defined by a genetic and transcriptomic background
Source: Nat Commun. 2018 Dec 4;9:5158. doi: 10.1038/s41467-018-07588-x (PMC6279830; doi:10.1038/s41467-018-07588-x)
Supplement: Supplementary file 2 — Description of Additional Supplementary Files [file 41467_2018_7588_MOESM2_ESM.docx]

**Description of Additional Supplementary Files**

**File Name:** Supplementary Data 1

**Description:** Environmental factors analysis of isolates transcriptome based on PCA. Results are summarized for the raw expression data, HPI and GAM adjusted data, top PCs adjusted data of w-GMS isolates and e-GMS isolates individually.

**File Name:** Supplementary Data 2

**Description:** *Tcvs* scores of 773 isolates/samples.

**File Name:** Supplementary Data 3

**Description:** Pathways enriched of genes showing expression convergence (red)/divergence (green) in geographical sites (GSEA p-value<0.05 & FDR<0.25)

**File Name:** Supplementary Data 4

**Description:** Pathways enriched of genes from same groups (*Grp1-6*) which were characterized in Fig. 2c.

**File Name:** Supplementary Data 5

**Description:** eQTL analysis in *e-*GMS parasites (p-value<1e-5).

**File Name:** Supplementary Data 6

**Description:** eQTL analysis in *w-*GMS parasites (p-value<1e-5).

**File Name:** Supplementary Data 7

**Description:** High confidence 5575 SNP-expression linkages which shows no contradictory associations between *e*-GMS and *w*-GMS parasites.

**File Name:** Supplementary Data 8

**Description:** Putative eQTL hotspots.

**File Name:** Supplementary Data 9

**Description:** Functional enrichment analysis of eQTL-regulated genes by eQTL/SNP type. Enriched pathways are defined at p-value<0.05 in hypergeometric test with gene number >=5. The yellow bar indicates the value of fold enrichment of gene frequency (observed/expected). The analysis is against MPM and GO pathways annotations which is listed separately in the table.

**File Name:** Supplementary Data 10

**Description:** Transcriptome-wide association study (TWAS) of artemisinin resistance in *e-*GMS parasites. The expression markers are defined at p-value<0.05 in Spearman’s *rho* test.

**File Name:** Supplementary Data 11

**Description:** Transcriptome-wide association study (TWAS) of artemisinin resistance in *w-*GMS parasites. The expression markers are defined at p-value<0.05 in Spearman’s *rho* test.

**File Name:** Supplementary Data 12

**Description:** Functional enrichment analysis of TWAS results. Enriched pathways are defined at p-value<0.05 and FDR<0.24 in GSEA with either or both *w-*GMS and *e-*GMS parasites.

**File Name:** Supplementary Data 13

**Description:** Artemisinin resistance associated eQTL linkages in *e-*GMS parasites.

**File Name: Supplementary Data 14**

**Description:** Artemisinin resistance associated eQTL linkages in *w-*GMS parasites.

**File Name:** Supplementary Data 15

**Description:** Raw expression values of 773 isolates transcriptome with sample IDs shown in columns and gene IDs in rows.

**File Name:** Supplementary Data 16

**Description:** HPI and GAM adjusted expression of 773 isolates transcriptome with sample IDs shown in columns and gene IDs in rows.

**File Name:** Supplementary Data 17

**Description:** Potential environmental factors involved in the 773 isolate samples including the known and estimated factors.

**File Name:** Supplementary Software 1

**Description:** R scripts of mixture model customized for HPI and GAM prediction in parasites at filed sites.
